# Does p-tau217 play a physiological reparative role in neonates and neurodegenerative diseases?

**DOI:** 10.1093/braincomms/fcaf321

**Published:** 2025-08-25

**Authors:** Timothy Daly, Lorenzo Pini, Bruno P Imbimbo

**Affiliations:** UMR 1219 Bordeaux Population Health, Université de Bordeaux & INSERM, Bordeaux 33076, France; UMR 5164, ImmunoConcept, University of Bordeaux & CNRS, Bordeaux 33076, France; Bioethics Program, FLACSO Argentina, Buenos Aires C1050 AAN, Argentina; Department of Neuroscience, University of Padova, Padova 35123, Italy; Padova Neuroscience Center, University of Padova, Padova 35129, Italy; Research & Development, Chiesi Farmaceutici, Parma 43122, Italy

We read with great interest the recent article by Gonzalez-Ortiz *et al*.^1^ in *Brain Communications*, which reported in multiple cohorts that plasma levels of phosphorylated tau217 (p-tau217) are highest in newborns, exceeding those observed in individuals with Alzheimer’s disease and in healthy subjects of any age. Notably, in a cohort of premature newborns, their data revealed a longitudinal decrease in blood-based p-tau217 levels during the first months of life, reaching baseline values similar to those found in young adults. The authors thus proposed a dual role for tau phosphorylation in newborns and in Alzheimer’s disease: protective against tau aggregation in early life and pathological in the context of Alzheimer’s disease. These findings can also be interpreted through the lens of brain activity and connectivity: elevated p-tau217 levels in newborns could reflect the increased activity characteristic of early development, whereas in Alzheimer’s disease, increased p-tau217 levels may rather represent a compensatory response to ongoing neuronal injury.

Tau protein is a highly abundant, microtubule-associated protein that is expressed primarily in neurons. It exists in three main states—non-phosphorylated, phosphorylated and aggregated^2^—and plays a dynamic role in stabilizing microtubules that determine cell shape and locomotion. Tau’s affinity for microtubules is modulated at over 50 sites by reversible phosphorylation, which allows for cytoskeletal disassembly and remodelling processes required for axonal transport, neurite outgrowth and activity-dependent synaptic plasticity.^3^ Phosphorylation also ensures that tau remains confined to axons under physiological conditions so as to further contribute to neuronal plasticity and organelle trafficking.^4^ However, our knowledge of tau function remains limited, as most tau studies have focused on the detergent-insoluble, aggregated form present in neurofibrillary tangles (NFTs), a pathological hallmark of Alzheimer’s disease, along with amyloid-β (Aβ) deposition.^3^

Gonzalez-Ortiz *et al*.^1^ consider ‘pathological tau aggregation and tangle formation’ to be a result of ‘abnormal phosphorylation [that] contributes significantly to disease progression’ in Alzheimer’s disease. However, since phosphorylation patterns in physiological and pathological tau are heterogeneous and overlapping,^3^ the authors propose a dual role for tau phosphorylation: during foetal development, it supports microtubule dynamics and neuroplasticity, whereas in Alzheimer’s disease, it may promote tau aggregation and tangle formation. Implicit in this view is the idea that a downstream pathological factor induces tau aggregation, rather than phosphorylation *per se* being inherently pathogenic.

Their hypothesis aligns with other findings showing increased phosphorylated tau levels in a range of neurological disorders, including traumatic brain injury.^5^ However, mechanistic studies in animal models challenge the causal interpretation of tau aggregation in neurodegeneration. Instead, the frequent observation of aggregated tau may reflect a form of survivorship bias, a cognitive fallacy in which conclusions are drawn from entities that have passed through a selection filter. Using two-photon longitudinal imaging, Zwang *et al*.^6^ recently demonstrated that neurons lacking NFTs died more than three times as often as those with NFTs and hypothesized that NFT formation may represent a mechanism of cellular senescence that protects neurons from apoptosis. Together, these findings suggest that elevated p-tau217 may reflect a compensatory or reparative response to both chronic and acute neuronal stress.

Further supporting this idea, a recent study comparing electroencephalogram patterns in 510 older adults across various stages of cognitive decline and 696 children at different stages of neurodevelopment showed that cognitive and functional decline in Alzheimer’s disease mirrors, in reverse, the developmental trajectory seen in childhood and adolescence.^7^ These findings are consistent with the retrogenesis hypothesis (RH), first proposed by Reisberg *et al*.,^8^ which posits that the order of cognitive decline in Alzheimer’s disease is the inverse of normal development: abilities gained in adolescence (e.g. abstract thinking) are lost first, followed by those acquired in childhood (e.g. performing basic tasks), and finally early childhood functions (e.g. walking, speaking). Behavioural, cognitive and language studies support this sequence. Neuroanatomically, RH aligns with the fact that Alzheimer’s disease preferentially affects brain regions that mature later in development, such as the hippocampus and association cortices. Although most neuroimaging studies have focused on older adults, similar ontogenic patterns have been reported in white matter development and in other neurodegenerative conditions such as frontotemporal dementia. In the final stage of the Global Deterioration Scale in late dementia, features include speech limited to one to five words, loss of intelligible vocabulary, complete loss of motor abilities, stupor and coma. Given the resemblance of these features with the functional profile of newborns, it is not surprising that neonates exhibit plasma p-tau217 levels that resemble, or are even higher than, those seen in Alzheimer’s disease patients. Elevated p-tau217 in both groups may reflect a biological adaptation of the brain to increased physiological or pathological demands.

But the parallels between early neurodevelopment and neurodegeneration extend beyond patterns of cognition. At the cellular level, both are shaped by activity-dependent remodelling processes. During development, the brain undergoes extensive structural refinement to sculpt efficient and specialized neural circuits, achieved through the selective, activity-dependent pruning of axons, dendrites and synapses formed in early life.^9^ These observations motivate the hypothesis that elevated p-tau217 in newborns may also participate in structural reorganization of brain circuits. Conversely, in neurodegenerative conditions such as Alzheimer’s disease, molecular pathways might be re-engaged maladaptively and so contribute to synapse loss. Hence, tau phosphorylation, adaptive in newborns, could well play a role in the aberrant reactivation of developmental programmes: in newborns, heightened activity could reflect a physiological drive for synaptic refinement and network specialization, whereas in Alzheimer’s disease, it may represent an unsustainable compensatory response to synaptic dysfunction and emerging neuronal stress. In both contexts, tau phosphorylation may serve as a molecular mediator of plasticity-related processes. Understanding these parallels between neurodevelopment and neurodegeneration may help clarify whether p-tau217 is a marker of neural resilience, vulnerability, or perhaps both, as a function of timing, cellular context, and functional state of the brain.

In conclusion, the more nuanced view of tau phosphorylation proposed by Gonzalez-Ortiz *et al*.^1^ should inspire a shift in the Alzheimer’s disease research field towards the study of normal tau physiology and its relationship with phosphorylation. Indeed, as Wegmann *et al*.^3^ note in the review cited by the authors: ‘Our current detailed understanding of Tau isoform in the CSF is contrasted by the lack of knowledge about the origin and function of these peptides’. That is, because of our decades-long focus on its role in pathology, we still lack a comprehensive mechanistic understanding of the physiology of tau protein. Scholars working on mechanistic explanations in neuroscience have long argued that stand-alone causal claims such as ‘C causes E’ are inferior to deeper mechanistic accounts that clarify how components interact in specific contexts to produce a given phenomenon.^10^ Thus, a more informative mechanistic approach would involve investigating the physiological roles of phosphorylated tau across the lifespan, elucidating the fine-grained mechanistic detail underlying relevant phenomena (e.g. the spatio-temporal interaction of p-tau217 with other entities that allow for development neuroplasticity) and then deriving causal inferences for pathology based on this more extensive mechanistic understanding. Instead of continuing with the prevailing approach that treats p-tau217 as inherently pathogenic within the faltering tau-reducing therapeutic strategy in Alzheimer’s disease, Gonzalez-Ortiz *et al*.’s^1^ findings invite new avenues for explanation and therapy in Alzheimer’s disease research.

## Data Availability

No new data were generated or analysed in support of this research.
